# Maxillofacial fractures among non-indigenous ethnic groups in the Irish national maxillofacial unit: a review

**DOI:** 10.1007/s11845-024-03681-x

**Published:** 2024-04-27

**Authors:** Liam Costello, Akinsola Ogunbowale, Kumara Ekanayake

**Affiliations:** 1 National Oral and Maxillofacial Unit, St. James‘ Hospital Dublin, Dublin, Ireland; 2 Dept. of Oral and Maxillofacial Surgery, University Hospital Limerick, Limerick, Ireland

**Keywords:** Injury, Maxillofacial, Non-indigenous, Trauma

## Abstract

**Background:**

This study investigates maxillofacial fractures in non-indigenous ethnic groups who were reviewed in the national maxillofacial unit in Ireland. The aim of this study was to highlight any potential trends in presentation of facial fractures in non-indigenous groups in comparison to previous reports which have included all ethnicities. This unique study is based on the fact that Ireland has only recently transformed into a diverse, multi-cultural country. This is unlike countries such as the UK and USA which have a long history of multicultural integration.

**Materials and methods:**

This retrospective study evaluated the trauma database of 4761 patients with 5038 fractures who attended the national maxillofacial unit over a 5-year period from 2015 to 2019. Parameters included age, gender, mechanism of injury, fracture sustained, time of the day, day of the week, month of injury, and the referral source were obtained from patient records.

**Results:**

The study identified 456 patients who did not identify as being born in Ireland, with 384 males and 72 females. The most common fracture seen was of the zygomatic bone, and the most common mechanism of injury was alleged assault for this cohort. Most injuries occurred in late afternoon with Friday being the most common day of the week.

**Conclusion:**

This study shows how maxillofacial units need to adapt to the changing trends in Irish demographics with increased demand for resources such as translation services. A further study could evaluate the rapidly changing demographic with mass migration of people currently seeking refuge in Western Europe.

## Introduction

Facial injuries comprise a large component of the day-to-day running of maxillofacial units. In addition to the increased workload they create, these injuries are also cause of significant physical and mental morbidity to affected patients.

Risk factors for sustaining maxillofacial injuries include male gender, interpersonal violence, and alcohol consumption, factors which often present in unison [1, 2]. The association of trauma with young males is well-documented in the literature with males comprising up to 67.8–89% [3–6] of patients in epidemiological studies.

The presentation of maxillofacial trauma differs worldwide with varying patterns of age ranges, gender, race, and geographical location. These variations are impacted by differences in local, regional, and national laws and customs [7].

In recent decades, Ireland has transformed into a diverse and multicultural society experiencing net inward migration, which rose from 8302 in 1996 to 153,881 in 2002 [8]. This compares to larger countries such as the UK, France, Belgium, and The Netherlands which have a longer history of multicultural diversity [9].

The most recent census figures from 2016 have shown that 11.6% of the Irish population are non-Irish nationals. This number had fallen from 12.8% in the previous census, partly due to an increase in the number of people obtaining Irish or dual citizenship [10] in addition to people returning to their country of origin.

Therefore, this study aimed to explore the epidemiology of maxillofacial fractures in the cohort of patients who identified as non-indigenous to Ireland, residing within the catchment area covered by the National Oral and Maxillofacial Unit.

## Materials and methods

Patients who presented with maxillofacial trauma were identified from a designated trauma database from the National Oral and Maxillofacial Unit at St James’s Hospital, Dublin, over the period of January 2015 to December 2019. Epidemiological data was gathered using electronic healthcare records with the addition of telephone follow-up for the completion of data. For the purpose of this study, non-indigenous was defined as those who emigrated to Ireland from countries excluding the UK and Western Europe. In addition, developed countries such as the USA, Australia, Canada, and New Zealand were excluded.

Epidemiological data of interest included gender, age, ethnic background, and duration of stay in Ireland. Additional data was obtained focusing on fracture sustained, single or multiple injuries, mechanism of injury, season of year at presentation (winter, summer spring and autumn), time of day (morning, afternoon, evening), the involvement of alcohol, and police intervention.

For those patients admitted, further data regarding surgical intervention and any associated complications post intervention were recorded.

Mechanisms of traumatic injury were subdivided into alleged assault (AA), animal involvement, domestic injury, fall, road traffic accident (RTA), sporting injury, and workplace injury.

The fractures were subcategorised into mandibular, zygomatic, Le Fort, frontal bone, nasal, and orbital wall fractures. Those with soft-tissue and dental-related injuries with no evidence of maxillofacial fracture were excluded from the study population.

The unit is a referral-centre for maxillofacial trauma with a geographical coverage of over 2.5 million inhabitants [11]. Of this population, Dublin City had the highest proportion of non-Irish nationals at 17.1%, this compared to Kilkenny (also covered by the unit) which had the lowest proportion at 8.1% in the census of 2016 [12].

As this is an epidemiological study, the focus remained on descriptive analysis demonstrating the relationship between the defined non-indigenous cohort of patients and the aforementioned categorical variables. The data was collected retrospectively, and statistical analysis was performed using IBM-SPSS 28 (IBM Corp., Armonk., N.Y., USA, 2021).

## Results

### Study population

A total of 4761 patients were identified over the 5 years, of which 456 (9.6%) were non-indigenous. There was a noted yearly increase in the number of those who sustained fractures in the study period. Males were disproportionately represented at 84.2% compared to females at 15.8% with, a ratio of 5.3:1 (Table 1).

**Table 1:** Yearly attendance for maxillofacial fractures from 2015 to 2019 with male and female breakdown

Year	Non-Indigenous (%)	Male (%)	Females (%)
2015	63 (13.8)	28 (7.3%)	34 (48.6)
2016	81 (17.8)	70 (18.2)	11 (15.3)
2017	81 (17.8)	81 (21.1)	0 (0)
2018	110 (24.1)	83 (24.2)	17 (23.6)
2019	121 (26.5)	112 (29.2)	9 (12.5)
**Total**	**456**	384 (84.2)	72 (15.8))

### Ethnicity

The highest percentage of patients accounting for over half of the cohort originated from Eastern Europe (52.4%). This may be due to its proximity to Ireland and freedom of movement enjoyed within the European Union (Table 2).

**Table 2:** Origin of patients and involvement of police as per ethnicity

	Non-indigenous (*n*)	%	Police involvement (*n*)	%
African	31	6.8	12	9.6
Eastern European	239	52.4	70	56
Far East	60	13.2	15	12
Middle East	84	18.4	17	13.6
North American	14	3.1	4	3.2
South American	18	3.9	3	2.4
South East Asia	10	2.2	4	3.2
**Total**	**456**		**125**	

### Duration of residence

**Fig. 1:**
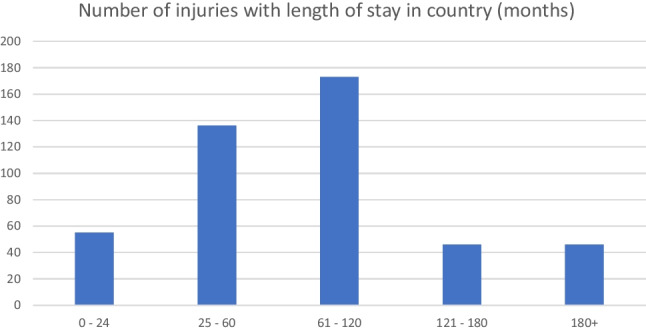
Length of stay in country (months)

The average duration of the stay in Ireland at the time of injury was 83.1 months. This ranged from approximately 1 to 279 months. The majority of injuries were sustained in those who stayed in the country between 5 and 10 years. (Fig. 1).

### Age

**Fig. 2:**
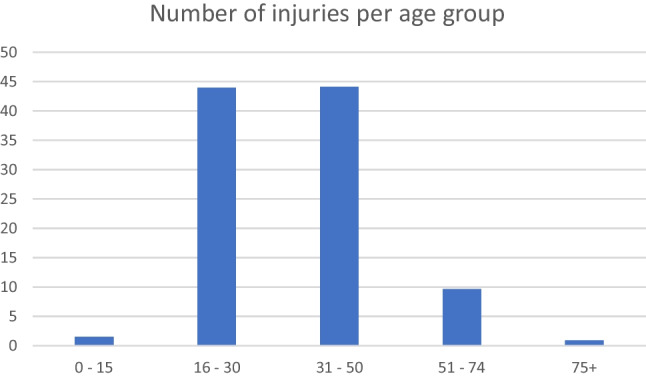
Distribution of injuries by age group

The average age of patients was 40.3 years, with a range of 1–100 years. The highest incidences of fractures occurred in the 16–30 (43.9%) and 31–50 (44.1%) age groups, as demonstrated in Fig. 2.

### Mechanism of injury

**Fig. 3:**
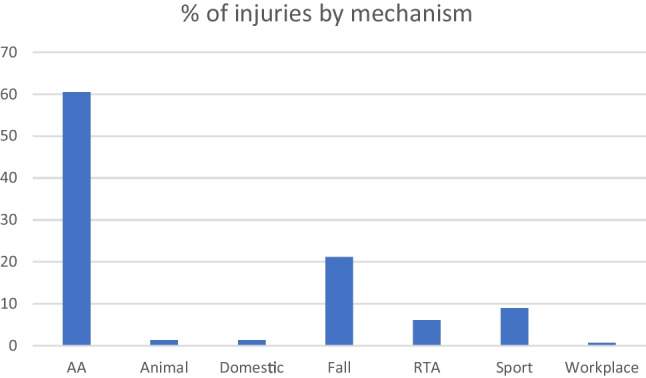
Distribution of injuries by mechanism

The overwhelming cause of injury was alleged assault with 276 patients (60.5%), followed by falls at 96 (21.1%) and sporting-related injuries 41 (9%) (Table 3). Injuries occurring in the workplace caused the least number of injuries in this cohort (Fig. 3).

**Table 3:** Mechanism of injury with relation to alcohol and residential location at the time

**Mechanism of injury**	**Numbers (%)**	**Alcohol involvement (%)**	**Inner city (%)**	**Outer city (%)**
AA	276 (60.5)	80 (76.9)	177 (58.03)	99 (65.6)
Fall	96 (21.1)	19 (18.3)	71 (23.3)	25 (16.5)
Sport	41 (9)	3 (2.9)	29 (9.5)	12 (7.9)
RTA	28 (6.1)	0 (0)	20 (6.6)	8 (5.3)
Animal	6 (1.3)	0 (0)	2 (0.65)	4 (2.6)
Domestic	6 (1.3)	2 (1.9)	5 (1.6)	1 (0.7)
Workplace	3 (0.7)	0 (0)	1 (0.32)	2 (1.4)
**Total**	**456**

### Pattern of injury

The anatomical distribution of injuries, shows 480 fractures occurring in the non-indigenous cohort.

Of the 480 fractures occurring in the non-indigenous cohort, 432 patients sustained a single fracture, and 24 patients sustained multiple fractures. The most common concurrent injury was to the nasal bone and orbital bones. The most common isolated injury was the fractured zygomatic complex bone 149 (31.04%), followed by nasal bones 112 (23.3%). The least common injury in this study was the Le-Fort pattern of fracture, with six cases seen during the study period.

### Day and time and season of injury

Most injuries during the week occurred on a Friday and Saturday, with 113 (24.8%) and 106 (23.2%) patients respectively. From those 2 days, a fracture of the zygomatic bone constituted the highest number at 34 and 35 respectively. The least amount of injuries occurred on a Monday with 40 (8.8%). A similar pattern observed with patients from both urban and rural areas.

Regarding the time of the day, most injuries occurred in the afternoon between 12:00 and 18:00 h and the least in the morning period (00:00–12:00 h). The average timing of the injury was 15:43 h with little discrepancy between the rural (15.2 h) and urban setting (15.5 h).

### Residential location/source

The majority of patients were referred from urban hospitals, (66.9%) compared to 33.1% from rural hospitals. For both cohorts of patients, alleged assault is the most common cause of the injury. The average age of the inner city and rural injuries were 34.7 and 34 years respectively. Nasal bone (29.6%) and zygomatic bone (28.3%) injuries are more common in inner city patients when compared to zygomatic fractures alone in the rural patient cohort (36.5%) (Table 4).

**Table 4:** Site of injury with relation to residential location, police involvement, alcohol involvement, and surgical intervention

	**Non-Indigenous (%)**	**Inner city (%)**	**Outer city (%)**	**Police involvement (%)**	**Alcohol involvement**	**Surgical intervention**
Zygomatic	149 (31.04)	91 (28.3)	58 (36.5)	43 (31.9)	35 (22.9)	44 (22.9)
Orbital walls	103 (21.5)	63 (19.6)	40 (25.1)	29 (21.5)	29 (18.9)	31 (16.1)
Nasal Bone	112 (23.3)	95 (29.6)	17 (10.7)	28 (20.7)	37 (24.2)	38 (19.8)
Mandible	98 (20.41)	61 (19)	37 (23.3)	30 (22.2)	49 (32)	70 (36.5)
Frontal Bone	12 (2.5)	6 (1.9)	6 (3.8)	3 (2.2)	0 (0)	5 (2.6)
Le Fort	6 (1.25)	5 (1.6)	1 (0.6)	2 (1.5)	3 (2)	4 (2.1)
**Total**	**480**	**321**	**159**	**135**	**153**	**192**

### Alcohol involvement

One hundred four (22.8%) patients admitted to having alcohol on board at the time of the injury, with 19 (18.3%) females and 85 (81.7%) males. It was not possible to record whether the assailant had consumed alcohol. Of those involved, alleged assault constituted 80 (76.9%) presentations and no cases of alcohol involvement were identified in road traffic accidents, workplace-related injuries, and animal-related injuries, as shown in (Table 3). Most alcohol-related injuries occurred on Friday and Saturday. Patients were most likely to fracture the mandible and nasal bones with alcohol involvement (Table 4).

### Police involvement

One hundred twenty-five patients in this study had police involvement, of which 100 cases were due to alleged assault (80%) with the remaining 25 associated with road traffic accidents. In cases of alleged assault, the assailant was known to the patient in 32 cases (26.6%). Among those involved in RTAs, 89.3% (25 out of 28 patients) cases had police involvement, likely due to the legalities surrounding RTAs and insurance purposes. Zygomatic bone fractures were the most common injury sustained when police were involved with 31.9% and, Le-Fort fracture the least (1.5%). Patients from rural hospitals constituted 36.8% of those with police involvement, as compared with 63.2% of patients from inner city hospital. Table 2 shows the ethnicity of those injured that the police were involved with 70 patients (56%) from Eastern Europe.

### Surgical intervention

In the 5-year period, 176 patients (38.6%) were admitted for a surgical intervention relating to their injury of which 35 patients were female and 141 were male. Sixteen patients sustained multiple injuries as compared to 160 patients who had a single isolated injury.

One hundred ninety-two procedures (Table 4) were carried out with reduction of the mandible accounting for the most cases at 36.5% (70) with the least being for frontal bone 2.1% (four) injuries. The average length of stay in the hospital was 2.05 days (ranges from 1 to 5 days).

Post-operative complications included those which occurred within 6 weeks of surgery. There were 17 (8.8%) complications noted out of the 192 surgeries performed. Of these, wound infection was accounted for the most at 23.6%. One patient suffered persistent paraesthesia of the mental nerve and 2 (11.8%) patients with non-union of their mandible fracture.

## Discussion

Injuries to the maxillofacial region are a common occurrence, mainly due to their prominence and vulnerability compared to other parts of the body. The aetiology of injuries to the face can vary greatly from one country to another and even within the same country depending on local laws and customs [1, 2].

The male-to-female ratio of the non-indigenous cohort mirrored that of other studies, with a very strong male preponderance due to association with risk factors such as IPV [3–5]. Males are also more likely to migrate, and this will in turn be reflected in the numbers involved in any injuries involving the maxillofacial region.

Over half of the patients in this study originated from Eastern Europe (52.4%), this compares to those from South East Asia (2.2%). This is likely to be due to the geographical proximity, in addition to the freedom of movement within Europe. Europeans tend to have a higher alcohol consumption rate when compared to Southeast Asian, African, and Middle Eastern populations [13]. This may also contribute to the over-representation of this cohort in the study.

This study shows that those living in Ireland between 2 and 10 years were more likely to sustain maxillofacial fractures. This compares to those who were new to the country and residing for more than 10 years. The reason for this large cohort may be due to assimilation into the local culture and environment, whereas individuals residing longer may be older in age.

Most of the facial fractures in the study were sustained in early adult life to middle age, with two peaks at 16–30 years and 31–50. Most studies have shown that facial injuries occur in a younger age group due to various activities associated with that stage of life, e.g., IPV and alcohol consumption.

In developed countries, the most common cause of maxillofacial injuries is interpersonal violence, whereas in developing regions, several studies have reported a strong association with road traffic accidents [3, 7, 14].

In our study, the most common mechanism of injury was interpersonal violence 60.5%, which in turn was followed by falls 21.1%. It is worth noting that while many of these patients are from low income countries, IPV remains the most common mechanism of fracture.

Fractures caused by RTA were only seen in 6.1% of patients; this may be a testament to the strict Irish road trafficking laws. A national survey conducted in 2017 showed 94% compliance with seatbelt usage nationwide [15]. A recent multicentre study from Israel showed that vehicle drivers also accounted for a small component of maxillofacial fractures nationally [16].

Whereas IPV was the most common mechanism of injury, the zygomatic-maxillary complex was the most common fracture sustained, followed by orbit and nasal bones. This suggests that trauma as a result of blunt force to the face as the most common cause of maxillofacial injury in this group.

Alcohol consumption was commonly associated with the cohort of patients who were involved in alleged assault (76.9%). Therefore, it is reasonable to suggest that IPV whilst intoxicated is the most prevalent mechanism of maxillofacial injury in the non-indigenous patient. To analyse this further, a comparison of patients native to Ireland presenting with maxillofacial injuries should be identified to highlight any potential differences or similarities to how patients present.

Overall, the consumption of alcohol was associated with 22.8% of patient presentations which echoes findings from a previous study by Ugboko et al. in a Nigerian population [17]. This confirms the known risk of alcohol consumption as an aetiological factor in maxillofacial injuries [18, 19]. It may be suggested that the rate of alcohol-related facial injuries is much higher than that we have recorded in this study, as it was not possible to accurately record the intoxication status of the assailant.

Most injuries in our study occurred on a Friday and Saturday, with (24.8%) and (23.2%) respectively. This is similar to many studies that show most injuries occurred at the weekends on both Friday [20, 21] and Sunday [18]. This trend is most likely to be observed due to increase in alcohol-related social interactions and activities, but also due to individuals undertaking sporting activities and car journeys, all of which have been seen as potential causes of maxillofacial fractures.

Monday saw the least amount of injuries occurring (8.8%), and a similar pattern was observed with patients from both urban and rural areas.

As 60.5% of the patients from this study sustained their injuries as a result of alleged assault, it would be reasonable to consider that motivation of assault may have racial implications. From this cohort, we found that police were involved in 125 cases where 100 patients sustained an injury as a result of assault. From these 100 patients, only 32% patients knew their assailant. This therefore highlights the thought that assault in the non-indigenous group may be motivated racially.

Fractures of the zygomatic complex were the most commonly affected bone in this study group at 31.04%, whereas mandibular fractures have been previously reported as the most common fracture pattern in other studies [1, 5]. The injury which occurred the least was fractures in a Le Fort pattern, with most Le-Fort fractures were caused by high-impact causes such as RTA, which as a mechanism of injury was low in our study.

When observing injuries sustained in urban or rural environments, patients had the same average age and injuries more commonly acquired as a result of interpersonal violence. A disparity existed between site of injury with nasal bone fractures occurring more common in urban settings and zygomatic fractures in more rural areas. These findings are similar to that of Batista et al., who found the same fracture presentation comparing urban and rural patient cohorts in a Brazilian population [19].

This study had several limitations; first being its retrospective nature which limited the scope of the data available from hospital records and patient charts. The size of the study population was also small with some nationalities underrepresented. There may also be some discrepancy where some patients identified as Irish but did not hold either an Irish passport or Irish citizenship.

## Conclusion

It is possible to recognise from this study that the numbers of patients identifying as non-indigenous to Ireland are growing year-on-year and the majority of injuries were related to inter personal violence with a high incidence of alcohol involvement. This may present challenges for the maxillofacial service with increasing demands for translating services and the availability of patient information leaflets in foreign languages. In the current climate of large-scale migration to Western Europe and beyond, there is scope for a prospective study to assess the rapidly changing demographics in Ireland and possible changing trends in the presentation of patients with facial injuries to emergency departments.
